# Influence of different scion-rootstock combinations on sugars, polyamines, antioxidants and malondialdehyde in grafted grapevines under arid conditions

**DOI:** 10.3389/fpls.2025.1559095

**Published:** 2025-06-27

**Authors:** Sonu Krishankumar, Jacobus J. Hunter, Mohammed Alyafei, Usama Souka, Sreeramanan Subramaniam, Ayyagari Ramlal, Shyam S. Kurup, Khaled M. A. Amiri

**Affiliations:** ^1^ Integrative Agriculture Department, College of Agriculture and Veterinary Medicine, UAE Uiversity (UAEU), Al Ain, United Arab Emirates; ^2^ ARC Infruitec-Nietvoorbij, Agricultural Research Council, Stellenbosch, South Africa; ^3^ Department of Nutrition and Health (CMHS), UAE University (UAEU), Al Ain, United Arab Emirates; ^4^ School of Biological Sciences, Universiti Sains Malaysia (USM), Georgetown, Penang, Malaysia; ^5^ Centre for Chemical Biology (CCB), Universiti Sains Malaysia (USM), Bayan Lepas, Penang, Malaysia; ^6^ Khalifa Center for Genetic Engineering and Biotechnology, UAE University (UAEU), Al Ain, United Arab Emirates; ^7^ Department of Biology, College of Science, UAE University (UAEU), Al Ain, United Arab Emirates

**Keywords:** grafted grapevines, malondialdehyde, polyamines, sugars, antioxidant enzymes, drought stress

## Abstract

**Introduction:**

Grapevines are an important and economically significant fruit plant that is cultivated worldwide. Most of the world’s emerging wine-growing regions are in arid or semi-arid regions that are severely affected by drought. Grafting has emerged as an effective strategy to enhance drought tolerance, but the influence of scion–rootstock combinations on key biochemical and antioxidant parameters under water deficit conditions is not fully understood.

**Method:**

The study investigates the effects of grafting on antioxidant enzymes, malondialdehyde (MDA), polyamines and sugar metabolism in five grafted grapevine combinations exposed to three irrigation levels to optimise sustainable grape production in the arid regions of the UAE. Leaf samples were analysed for polyamines, MDA and sugars using liquid chromatography.

**Results:**

The study found that the drought-tolerant rootstocks Paulsen, R110 and Ramsey increased cell activity, reduced ROS production, lowered MDA levels and increased antioxidant capabilities. SOD activity increased significantly under 75% and 50% FC by 225% in V1, 316% in V3, 133% in V4 and 1025% in V5. In response to severe drought at 75% and 50% FC, V2 and V5 showed a decrease in MDA accumulation (by 34.2% and 50% in V2 and 23.7% and 57.6% in V5, respectively) compared to 100% FC (0.76 nmoL mL−1 in V2 and 1.18 nmoL mL−1 in V5). Polyamines generally showed an increase with decreasing field capacity, indicating drought tolerance. Different polyamine contents were observed in grafted vines. V1, V2 and V5 showed higher levels of spermine and free spermidine, while V3 and V4 were more sensitive to drought stress. Organic osmolytes positively influenced the enzymatic activity in drought-tolerant grafts. Sugars built up in the grafts and had a signalling function as Osmo protective molecules. The shoots improved sugar metabolism, which led to increased resistance to drought. There was a significant increase in glucose sugar content at 75% and 50% FC, which was 85.7% to 133% at V1, 19% to 76.9% at V2, and decreased by 30% and 53% at V4.

**Discussion:**

The results suggest that grafted grapevines have a strong ability to cope with drought stress by upregulating antioxidant enzymes and altering other compounds such as MDA, PA and sugars that are conducive to stress tolerance.

## Graphical Abstract

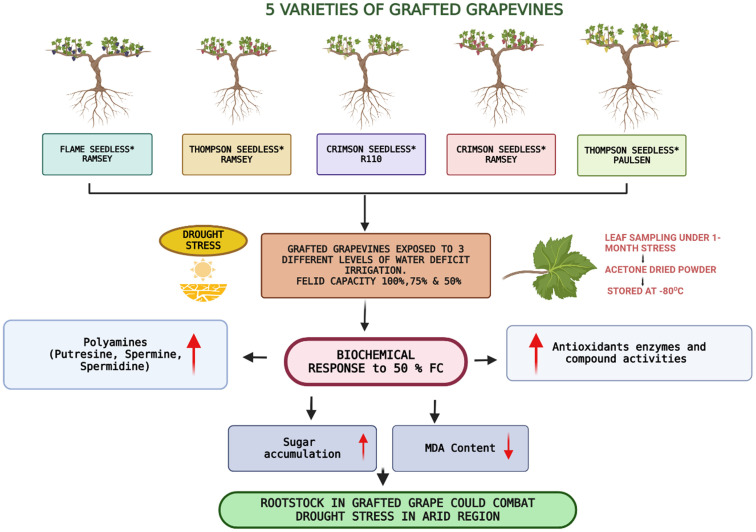

## 1 Introduction

The grapevine is an extensively grown fruit crop all over the world and has important economic value (Grassi and De Lorenzis, 2021). Many of the world’s emerging grape-growing regions are in semi-arid or dry areas that are greatly affected by drought (Seleiman et al., 2021). Among all abiotic stresses, drought is the most serious and complicated and has a severe adverse impact on plant growth and yield. It causes plant dehydration, which results from an imbalance between water absorbed from the substrate and water lost by transpiration (Aroca et al., 2011).

Drought stress significantly impacts the physiological and biochemical performance of grapevines, influencing properties and processes such as stomatal conductance, photosynthesis, and chlorophyll fluorescence leading to different behavior in different varieties (Dinis et al., 2014), and consequently affecting gene expression, reactive oxygen species (ROS) levels, and the overall defense response of plants (Haider et al., 2017; Zamorano et al., 2021).

To minimize the effects of stress, plants have evolved various adaptive mechanisms to maintain growth and productivity (Skirycz and Inzé, 2010). Grapevines respond to drought stress by modulating the phenolic compounds and antioxidants in the roots (Weidner et al., 2009). Under drought stress, plants lose their cell turgidity and overproduce ROS such as superoxide (O_2_^−^) and hydrogen peroxide (H_2_O_2_), which leads to oxidative stress. ROS can be scavenged through a series of antioxidant enzymes, such as superoxide dismutase (SOD), peroxidase (POD), and catalase (CAT) to maintain osmotic balance (Salazar-Parra et al., 2012; Bernardo et al., 2017; Wang et al., 2019). Capell et al. (2004) investigated the role of polyamines (putrescine, spermidine, and spermine) under drought stress conditions to protect plants in response to osmotic stress. Scholars have also revealed the ability of polyamines to reduce chlorophyll breakdown and enhance DNA synthesis under drought conditions (Liu et al., 2017). Research has shown that the interaction between polyamines and antioxidant enzymes under drought stress is crucial for plant stress responses as it can modulate stress-triggered ROS homeostasis and oxidative damage by activating antioxidant enzyme activities (Hong et al., 2013). To assess the degree of damage to plasma membranes and the capacity of plants to withstand drought stress, malondialdehyde (MDA) a substance produced by membrane lipids in response to ROS, can be used as a drought indicator (Laxa et al., 2019). Lin et al. (2023) highlighted the importance of defense responses, focusing on photosynthesis, antioxidant systems, and osmotic regulation, all of which help in maintaining plant homeostasis under water scarcity. The results indicated the potential link between antioxidant capacity, MDA levels, and drought tolerance in grapevines.

In viticulture, the grafting of scions onto rootstocks was originally used to combat the phylloxera disease, a soil-dwelling insect that wiped out large surfaces of European vineyards (Mudge et al., 2009). Grafting is a crucial approach that also helps grapevines to grow in more arid conditions and become more drought-tolerant and water-use efficient (Caruso et al., 2023; Bonarota et al., 2024). Grapevines can use drought-tolerant rootstocks to adapt to water constraints in areas with restricted water availability, such as desert environments (Azorín and García, 2020). Hence, to mitigate the negative effects of drought on grape production and quality, grafting is essential. To achieve sustainable grape production, it is necessary to understand how grapevines are affected by different rootstocks and how drought stress is tolerated physiologically and biochemically by different graft combinations.

Grafting is reported to regulate the synthesis of protective proteins, sugars, proline, and antioxidants in grapevines, enhancing their drought tolerance (Pagliarani et al., 2017). Tsegay et al. (2014) examined the responses of grapevine rootstocks to drought stress and demonstrated that drought-tolerant rootstocks exhibited enhanced water uptake and root growth during the dry season, contributing to improved water-use efficiency and drought tolerance in grapevines. de Oliveira et al. (2020) determined rootstocks as essential for helping vines adjust to unfavourable soil conditions including salinity, alkalinity, acidity, and fluctuations in water availability. Rootstocks can regulate secondary metabolism, including the production of sugars and phenolic compounds, which are crucial for grape quality and stress response (Zombardo et al., 2020). Drought-tolerant rootstocks in grapevines can significantly impact the antioxidant system of plants. Jiao et al. (2023) found that grafting onto the 1103 Paulsen rootstock improved the antioxidant defense capacity, thereby enhancing tolerance against drought stress in grapevines.

To ensure sustainable grape production in arid regions, selecting appropriate rootstocks is extremely important. Research has shown that different rootstocks can impact numerous aspects of grape development. For example, the rootstocks Dog Ridge, 99R, 110R, and 1103P were identified to have high phenolic content, potentially reducing grape diseases of the scion cultivar following grafting (Satisha et al., 2007). Nazir et al. (2022) determined that the 1103 Paulsen rootstock, originating from the cross of wild V. rupestris and V. berlandieri species, could efficiently reduce the effects of drought stress on grafted scion varieties by activating the differentially expressed genes that assist in water and nutrient uptake. Ramsey rootstock (*V. champinii*) has been linked to higher water-use efficiency and relative water content, maintaining higher photosynthesis levels to overcome drought stress in grapevines (Cochetel et al., 2020).

Understanding the impact of different rootstocks on drought tolerance mechanisms is essential for the sustainable production of grapes in arid regions such as the UAE. Hence, in this study, the antioxidant enzymes, MDA, polyamine, and sugar contents of five grafted grapevine combinations exposed to three levels of irrigation under field conditions were determined to gain insight into the requirements for sustainable grape production in arid regions.

## 2 Materials and methods

### 2.1 Meteorological data in Alain, Abu Dhabi, UAE

**Figure 1:**
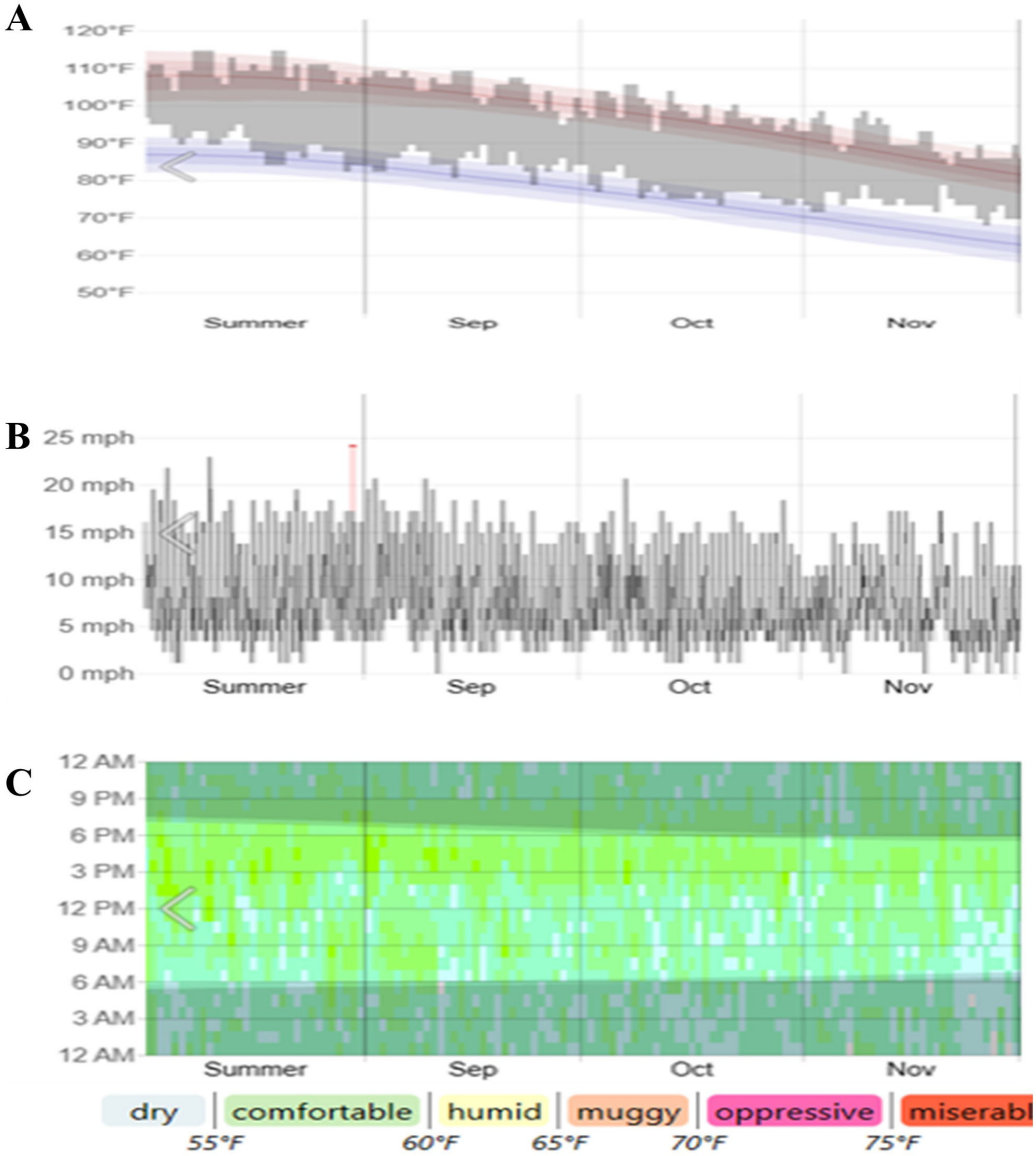
Temperature **(A)**, wind speed **(B)** and humidity data **(C)** (2022) from AL-Ain, Abu Dhabi, UAE. (Courtesy: Data from Abu Dhabi Meteorological Department).

Temperature, Wind speed, Humidity (2022) from AL-Ain, Abu Dhabi -UAE. (Courtesy: Data from Abu Dhabi Meteorological Department) (**Figures 1A–C**). Temperature, humidity, and wind speed are the primary factors influencing crops in terms of drought tolerance, and their optimal levels contribute to maintaining tissue water content. In arid regions, these parameters enhance the evapotranspiration rate, leading to complex drought effects. This study employed data for 3 months, including 1 month before drought induction and 1-month post-induction (September, October, and November 2022) (**Figure 1**).

### 2.2 Plant material and experimental site

Whip-grafted grapevine stocks were procured from South Africa in collaboration with ARC Infruitec-Nietvoorbij, Stellenbosch, South Africa. The rootstocks used for grafting were selected for extreme environmental conditions. Three table grape varieties, namely Thomson seedless, Crimson seedless, and Flame seedless, were grafted onto the drought-tolerant rootstocks Ramsey, 110 Richter (R110), and 1103 Paulsen (P1103). The five graft combinations used for the experiment were as follows: Flame seedless × Ramsey (V1); Thompson seedless × Ramsey (V2); Crimson Seedless × R110 (V3); Crimson Seedless × Ramsey (V4); and Thompson Seedless × P1103 (V5).

**Figure 2:**
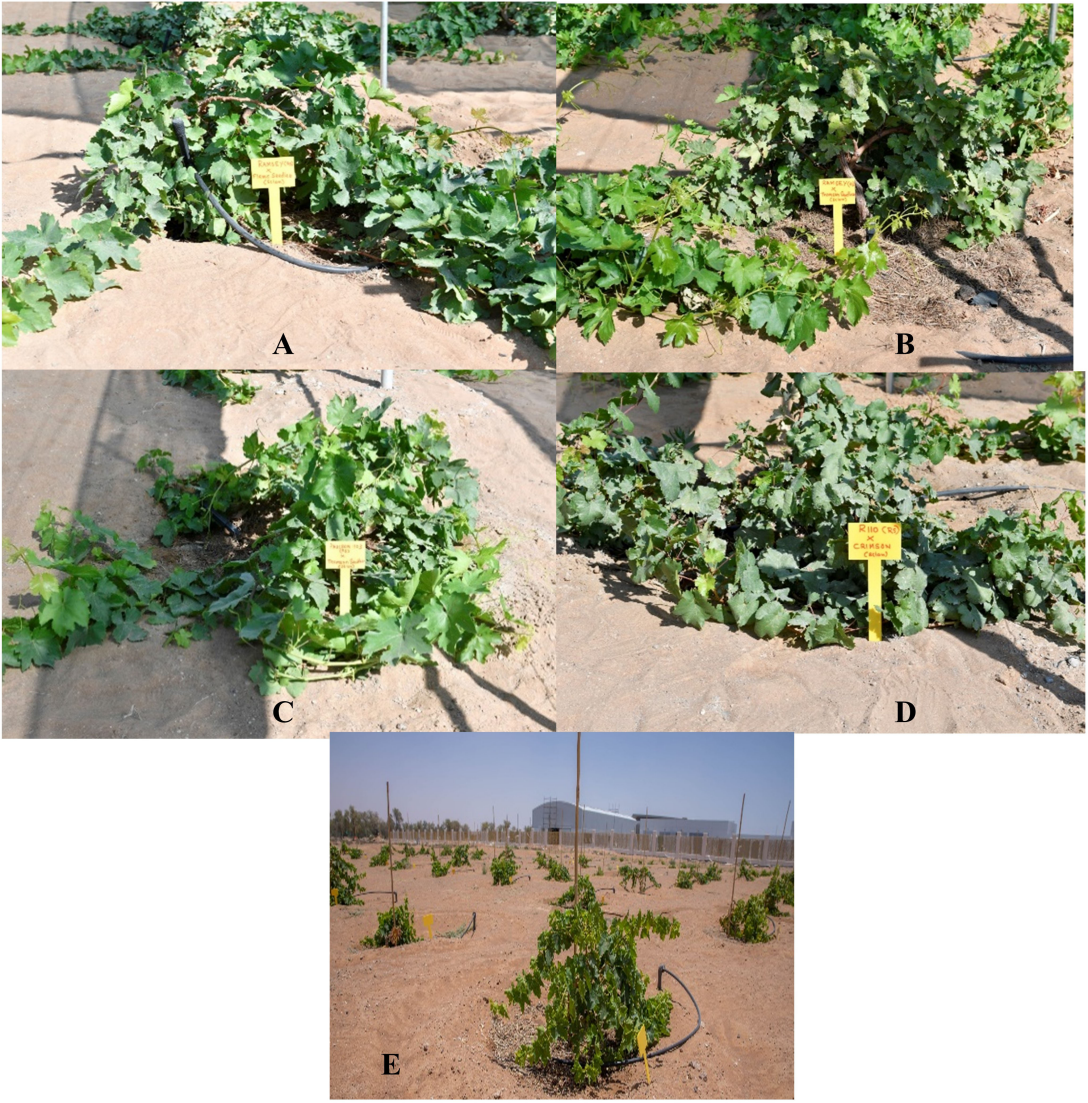
**(A)** Flame seedless grafted on to R.S. Ramsey (early stage of spreading), **(B)** Thompson seedless grafted on to R.S. Ramsey, **(C)** Thompson seedless grafted on to R.S. Paulsen (P1103), **(D)** Crimson seedless grafted on to R.S. R110 and **(E)** under different regimes of irrigation IW/CPE ratio of 1, 0.75 and 0.5 to induce drought.

The study was conducted at the College of Agriculture and Veterinary Medicine (UAEU) Experimental Farm, Al Foah, UAE, in open field conditions with complete exposure to natural light. Prior to planting in the field, the grafts were maintained under greenhouse conditions. After receiving the grafts, the grape vines were washed in normal water, drenched in fungicidal solution (Ridomil 1.5 g L^−1^), and potted in polyethylene bags filled with a substrate consisting of sterile sand, dehydrated cow manure, and peat moss (1:1:1) for bud initiation and acclimation. The grapevines were irrigated by the drip method to maintain optimal soil moisture for survival and growth. The greenhouse conditions included extra light consisting of a 16/8 h light/dark photoperiod (daily photoperiodic light–dark ratio of around 600 µmol m^−2^ s^−1^, temperature range of 23–28°C with a cooling system, and 65–80% relative humidity. After five months in the greenhouse, field planting was conducted in January 2022. The young vines were planted in planting pits measuring 60 × 60 × 60 cm at a spacing of 3 m (row spacing) × 2.5 m (vine spacing) and trained onto a flat Trentina trellis system with a north-to-south orientation for the rows (**Figure 2E**).

### 2.3 Experimental design

The experiment was performed using a factorial randomized complete block design. The graft establishment was successful within approximately 3 months, achieving 90% establishment. Gap filling was conducted with new grafts before irrigation treatment commenced. The experiment was divided into three irrigation regimes, with nine replicates for each graft combination. The irrigation levels were as follows: control, 100% field capacity [FC]); severe deficit (WS-S; 50% FC); and intermediate deficit (WS-I; 75% FC) with controlled drip irrigation. Stress was applied for 1 month (1–31 October 2022) and the grapevine leaves medium-matured with fully expanded lamina were collected for analysis from the middle of the vines in the 1st week of November and stored at −80°C until analysis. The vines approached the reproductive phase (started flowering) a month after sampling.

### 2.4 Irrigation treatments

A 200 g soil sample was collected, saturated with water, and maintained under a pressure of 100 psi for 24 h to extract the water held within the soil. Following this, the soil was collected, weighed, incubated for 48 h, and subsequently dried at 65°C in an oven. The weight of the dry soil was measured, and the soil moisture percentage was calculated (**Table 1**). Based on the IW/PE ratios of 1, 0.75, and 0.5, the calculations for 100% FC, 75% FC, and 50% FC were established. Irrigation at 53 L, 40 L, and 26 L was calculated for each plant based on the FC. To achieve the volume of water required per plant, drip irrigation was supplied in the morning and evening. The soil moisture was monitored to determine the induction of drought during deficit irrigation.

**Table 1:** Drought classification based on SWD1, VC1, and SM in an arid region.

Drought Classification	SWD1 Soil wetness deficit Index	VC1 (%) Vegetative condition Index	SM (%) Soil Moisture
No Drought IW/CP (1)	≥ 0	40 – 100	≥ 40 %
Mild Drought IW/CP (0.75)	0 to -2	30 – 40	≤ 40 %
Moderate to severe Drought IW/CP (0.50)	-2 to -5	20 – 30	≤ 30 %

SWD1, Soil Wetness Deficit Index; VC1, Vegetative Condition Index; SM, Soil Moisture.

### 2.5 Soil moisture

The soil moisture parameters were assessed to determine the extent of drought under various irrigation conditions (Zhou et al., 2021) (**Table 1**).

### 2.6 Biochemical analysis

#### 2.6.1 Leaf sample preparation

The grape leaves were cut into small pieces and ground in a pestle and mortar with liquid nitrogen. The powder was depigmented using acetone and maintained at −80°C for further analysis.

#### 2.6.2 Analysis of leaf sugar content by high-pressure liquid chromatography

The extraction of sugars was performed using the method described by Du et al. (2020).

##### 2.6.2.1 Sample preparation

A sample of 0.5 g of depigmented leaf powder was weighed into a 15 mL centrifuge tube and 10 mL of extraction solvent (80:20 acetonitrile: H_2_O, v/v) was then added. After 3 min of incubation at 80°C in a water bath, the mixture was shaken in an orbital shaker for 30 min. It was then centrifuged at 10,000 rpm at 4°C for 10 min. A PVDF (Millipore, Merck, USA) filter with a pore size of 0.45 μm was used to filter the supernatant before injection into a high-performance liquid chromatography (HPLC) system (refer to section 2.10).

##### 2.6.2.2 High-performance liquid chromatography conditions

**Figure 3:**
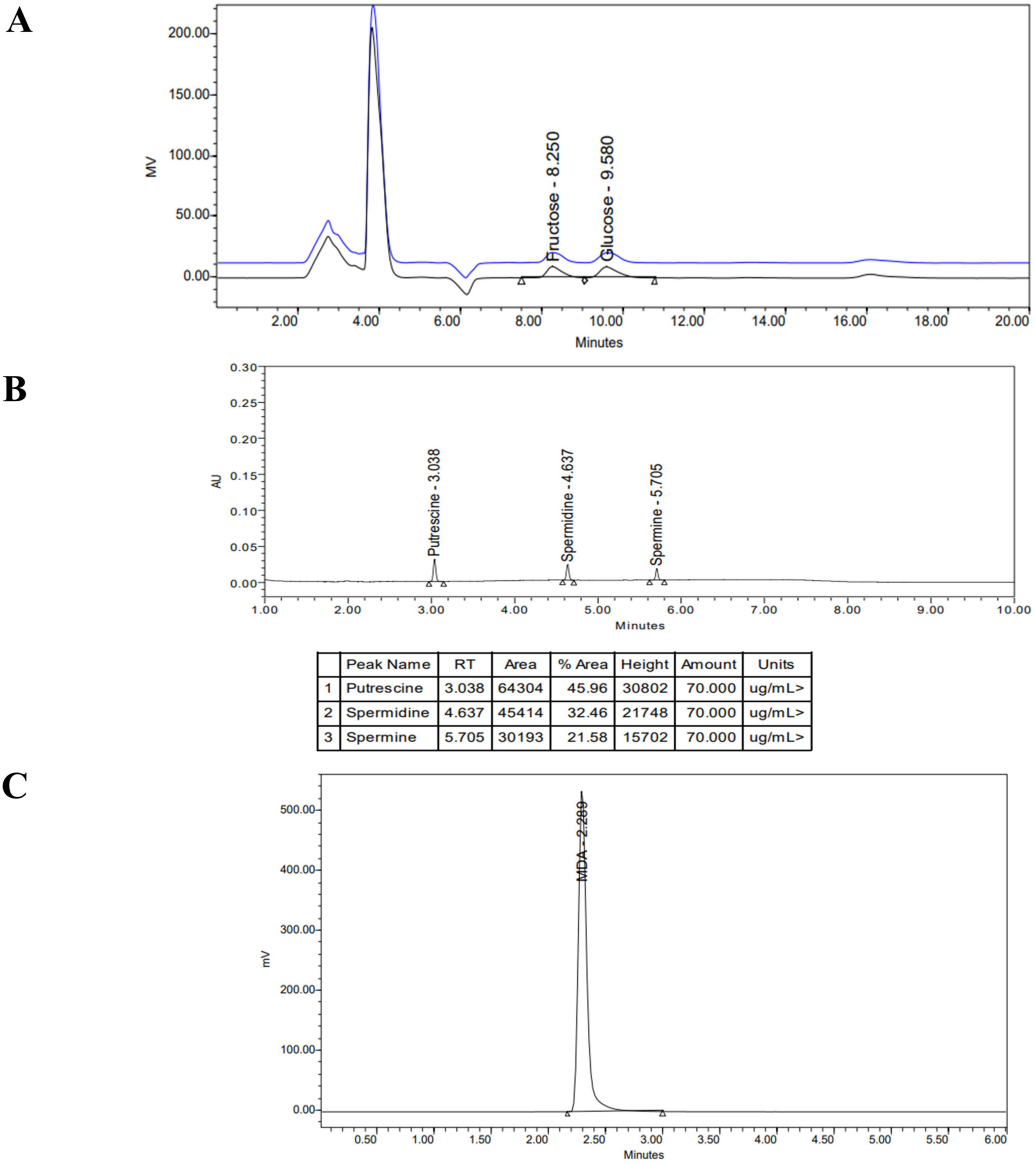
HPLC separation of **(A)** fructose & glucose, **(B)** polyamines, **(C)** MDA.

The contents of glucose and fructose were measured by HPLC (Waters 1525, USA) using an X-Bridge BEH Amide 250 × 4.6 mm column (**Figure 3A**). The analysis was performed at 35°C with a flow rate of 1 mL min^−1^ using isocratic elution with 75% acetonitrile (ACN):25% H_2_O and 0.2% tri ethyl amine as a mobile phase. Sugar content was measured in milligrams (mg) per gram of dry leaf weight using a standard curve.

#### 2.6.3 Polyamine analysis

All solvents were of analytical reagent grade. Standards of putrescine, spermine, and spermidine were purchased from Labco (FLUKA) and were of 99.0% purity. Dansyl chloride of LC grade was purchased from Sigma Aldrich with a purity of 99.0%. Chromatographically pure acetonitrile, acetone, ammonium acetate and AR grade ammonia, sodium bicarbonate, hydrochloric acid, and perchloric acid were obtained from Sigma.

##### 2.6.3.1 Preparation of standard solutions

Endogenous free polyamines were derived and analysed by ultra-pressure liquid chromatography (UPLC) according to the method of Wu et al. (2020), with minor modifications in the mobile phase. For the biogenic amine single-stock solutions (1 mg mL^−1^), standards of putrescine, spermine, and spermidine (100 mg each) were transferred into 100 mL volumetric flasks, respectively. Hydrochloric acid was added to each flask and mixed well to achieve 0.1 mol L^−1^ volume. For the mixed stock solution of three standard biogenic amines (100 μg mL^−1^), a 1 mL aliquot of each biogenic amine single-standard stock solution was pipetted into a 10 mL volumetric flask. Hydrochloric acid was added to each flask and mixed well to achieve 0.1 mol L^−1^ volume. For the derivatization of biogenic amines standard solution, a 1 mL aliquot of the combined stock solution (100 μg/mL) was pipetted into a 15 mL centrifuge tube, to which 1.5 mL of saturated sodium carbonate solution and 1 mL of dansyl chloride solution (10 mg mL^−1^, dissolved in acetone) were added. The tube was covered and shaken for 1 hr at 40°C in a temperature-controlled shaker without light. Following this, the process was stopped by adding 100 μL of ammonia. The volume was adjusted to 5 mL using acetonitrile, mixed well, and centrifuged for 5 min at 4000 rpm. Finally, a 0.22 μm PVDF filter was used to filter the supernatant in preparation for liquid chromatography.

##### 2.6.3.2 Preparation of the samples for UPLC-PDA analysis

A 10 mL centrifuge tube containing approximately 0.3 g of depigmented leaves was filled with 5 mL of 0.4 mol L^−1^ perchloric acid, vortexed, and centrifuged at 8,000 rpm for 10 min at 4°C. After the supernatant was pipetted, a 15 mL centrifuge tube was filled with an aliquot of 1 mL supernatant, 1.50 mL of saturated sodium bicarbonate solution, and 1.00 mL of dansyl chloride solution (10 mg mL^−1^, dissolved in acetone). The tube was then covered and placed in a temperature-controlled shaker set at 40°C, protecting it from light. Following this, a 100 μL dose of ammonia was added to terminate the derivative reaction. Acetonitrile was used to dilute the reaction liquid to 5.00 mL, and it was centrifuged at 4,000 r min^−1^ for 5 mins. A 0.22 μm PVDF filter was then employed to filter the supernatant for further analysis.

##### 2.6.3.3 UPLC conditions

An UPLC system (Waters, USA) was used to separate and analyse the content of the PAs with a BEH C18 (1.7um, 2.1 mm × 100 mm) reverse-phase column set at 35°C (**Figure 3B**). The mobile phases were A, water; and B, acetonitrile, with a flow rate of 1.0 mL min^−1^ (**Table 2**). Spectra were obtained for the three amines at 254 nm. Polyamine values were expressed in µg mL^−1^ (DW).

**Table 2:** Gradient table of mobile phase A and mobile phase B.

Time (min)	Mobile phase A %	Mobile phase B %
0	45	55
5	5	95
6	5	95
7	45	55
8	45	55

#### 2.6.4 Malondialdehyde measurements

The MDA concentration was determined following the method described by Morales et al. (2021), with minor modifications. Briefly, 0.2 g of depigmented leaf powder was transferred to a 15 mL centrifuge tube after weighing. Following this, 2 mL of 1 PPM butylated methanol was added, and the contents vortexed, sonicated for 30 min and centrifuged. The supernatant was collected and 400 µl of supernatant was transferred into 400 µl of 5% trichloroacetic acid (TCA), incubated in an ice bath for 20 mins, and centrifuged at 11,000 rpm for 15 mins. An aliquot of 400 µL of the supernatant was added to a centrifuge tube with a solution containing 400 µL of thiobarbituric acid (TBA) and incubated for 60 mins at 60°C. A total of 800 µL of butanol was then added and the sample was vortexed.

##### 2.6.4.1 HPLC conditions and mobile phase preparation

The samples were analyzed using a Breeze HPLC system (Waters Corporation, USA) including a 1525 pump, a 2475 multi λ fluorescence detector, a 717-auto sampler, and a system controller with a PC control program (**Figure 3C**) and a XTerra MS C18, 5 µm pore size reversed-phase column (4.6 ×150 mm) (Waters USA). The mobile phase consisted of a methanol–0.05 M KH_2_PO_4_ buffer with a pH 6.8 (40:60, v/v), containing 0.2% (v/v) tri-ethanolamine, implemented at a flow rate of 1 mL min^−1^ and column temperature of 35°C. The fluorescence detector wavelength was set at 532 nm (excitation) and 553 nm (emission) and the injection volume was 20 µl. The MDA concentration was expressed as nmol mL^−1^ based on dry weight.

#### 2.6.5 Determination of antioxidant enzyme activities

Antioxidant enzyme (SOD, CAT, POD) activity was determined as described by Abassi et al. (1998), with slight modifications. Two fully expanded leaves from nine plants (each plant is a replicate) were collected from each treatment. Briefly, a 1 g leaf sample was frozen in liquid nitrogen and ground in 10 mL extraction buffer. The ground leaf tissue was suspended in 15 mL of 100 mM KPO_4_ buffer (pH 7.8) and containing 0.5% v/v Triton X-100 and 1 g PVPP. The mixture was dialyzed overnight at 58°C against the same buffer and centrifuged at 10,000 rpm for 30 min at 48°C. The supernatant was collected for further analysis.

For SOD activity, a test mixture (300 μL of enzyme extract, 50 mM KPO4 buffer (pH 7.0), 200 μL of NBT + EDTA + methionine buffer, and 50 μL of riboflavin stock) was added to a 3 mL cuvette and was left in the dark at room temperature for 10 min. A spectrophotometer was used to measure the absorbance at 560 nm. SOD activity was expressed as µg^−1^ protein.

For CAT activity, an assay mixture (300 μL) of enzyme extract, 2.6 mL of 50 mM KPO_4_ buffer (pH 7.0), and 0.4 mL of 15 mM H_2_O_2_ were added into 3 mL cuvettes and placed in the dark at room temperature for 10 min. The absorbance was read at 240 nm after 45 and 60 sec using a spectrophotometer. COD was expressed as µg^−1^ protein.

For POD activity, an assay mixture was prepared consisting of 2.6 mL 15 mM NaKPO_4_ buffer (pH 6.0) and 100 μL enzyme extract, 0.15–0.15 mL 1 mM H_2_O_2_, and 0.1 mM guaiacol (o-methoxyphenol). The assay mixture absorbance was read at 470 nm with a spectrophotometer. POD activity was calculated over a 3 min period and expressed as µg^−1^ protein.

Ascorbate peroxidase (APX) activity was determined following the method of Nakano and Asada (1981). Briefly, a total volume of 2 mL of reaction mixture was pipetted into 3 mL glass cuvettes. The assay reaction mixture comprised 25 mM (pH 7.0) sodium phosphate buffer, 0.1 mM EDTA, 0.25 mM ascorbate, 1.0 mM H_2_O_2_, and 100 μL enzyme extract. The H_2_O_2_-dependent oxidation of ascorbate was monitored. The decrease in absorbance was read at 290 nm at 60-sec intervals for 10 min by a spectrophotometer and expressed as µg^−1^ protein.

Glutathione reductase (GR) activity was determined using the Foyer and Halliwell technique (Shehata et al., 2022). The reaction mixture consisted of 25 mM KPO_4_ buffer (pH 7.0), 0.1 mM EDTA, 0.5 mM oxidized glutathione (GSSG), 0.12 mM NADPH, and 0.1 mL enzyme extract at a total volume of 2 mL. GR activity was assessed by measuring the drop-in absorbance at 340 nm caused by NADPH oxidation for 30 sec.

The chromatogram represented for each analysis (MDA, sugars, polyamines) is to demonstrate peak separation, retention time, and compound identification.

#### 2.6.6 Relative water content

Relative water content (RWC) was determined using the 7–9^th^ node from the bottom of the grapevine leaves (Jiao et al., 2023). Following the drought treatment, grapevine leaves were collected, stored in dark containers, and transferred to the laboratory to determine the fresh weight (FW). The turgid weight (TW) was determined after the leaves were immersed in distilled water in the dark for 24 h at 4°C. The samples were dried at 105°C for 30 min and then at 80°C until their weight remained constant. The dry weight (DW) was then calculated. The RWC (%) was determined by Equation 1.


(1) $$\text{RWC}=\frac{\text{FW}-\text{DW}}{\text{TW}-\text{DW}}\times 100$$

### 2.7 Statistical analysis

A two-way analysis of variance (ANOVA) was performed using the general linear model procedure to calculate the effects of different irrigation treatments and graft varieties on amino acids. Means were compared using the Tukey HSD *post hoc* comparison test (P ≤ 0.05) using the Minitab statistical software (Minitab^®^ 21.4.3). Figures were plotted by using Minitab (Minitab^®^ 21.4.3) and Design Expert (version 13.0.5.0) (**Tables 2**, **3**; **Supplementary Table 1**).

**Table 3:** Summary of mean values of interaction effect from two-way ANOVA.

IRRIGATION LEVELS * VARIETIES	100% FC					75% FC					50% FC				
	V1	V2	V3	V4	V5	V1	V2	V3	V4	V5	V1	V2	V3	V4	V5
Glycine Betaine (GB)	122.2	86.8	122.6	128.2	133.4	134.8	123.0	140.6	143.4	191.0	287.6	319.8	350.8	294.8	387
Catalase (CAT)	7.6	6.4	4.6	6.8	11	11.4	9.2	9.0	12.2	16.6	16.8	11.6	12.6	16.2	27.8
Superoxide Dismutase (SOD)	13.4	17.0	12.0	16.6	7.8	18.2	17.0	12.6	18.0	17.4	52.8	31.4	50.8	43.4	89.0
Relative water content (RWC)	93.6	94.8	95.2	96.3	91.3	92.2	89.2	93.1	93.0	87.0	87.1	85.3	84.2	88.3	79.2
Peroxidase (POD)	8.8	12.6	6.8	11.0	8.2	11.2	35.0	13.8	13.0	13.4	32.2	32.6	38.0	32.0	45.8
Glutathione Reductase (GR)	7.8	4.2	7.4	10.4	9.8	8.8	7.8	10.8	10.8	10.4	16.0	17.2	19.4	22.0	21.0
Ascorbate Peroxidase (APX)	2.8	4.1	1.6	3.1	3.9	4.2	4.8	4.1	4.1	6.4	6.2	8.3	7.8	7.9	11.3
MDA	0.664	0.742	0.533	0.687	1.133	0.743	0.538	0.248	1.179	0.939	0.29	0.328	0.309	1.375	0.509
Putrescine	597.1	0	461.9	1008.1	453.6	776.3	0	409.5	379.3	467.9	422.6	0	0	308.8	442.7
Spermidine	564.1	139.6	611.8	759.9	473.3	759.0	206.26	617.9	448.5	604.0	863.2	208.7	40.2	350.4	598.2
Spermine	636.8	264.6	883.7	365.3	479.3	605.4	520.4	234.5	212.8	662.5	726.2	329.1	235.9	191.9	598.8
Fructose	21.5	26.5	37.5	43.1	45.2	40.2	29.5	38.6	29.7	31.5	49.0	36.4	23.2	20.6	44.4
Glucose	25.3	30.4	45.8	46.9	48.4	44.6	33.31	48.3	35.7	34.1	54.3	41.2	27.3	26.4	52.2

## 3 Results

### 3.1 Polyamines

**Figure 4:**
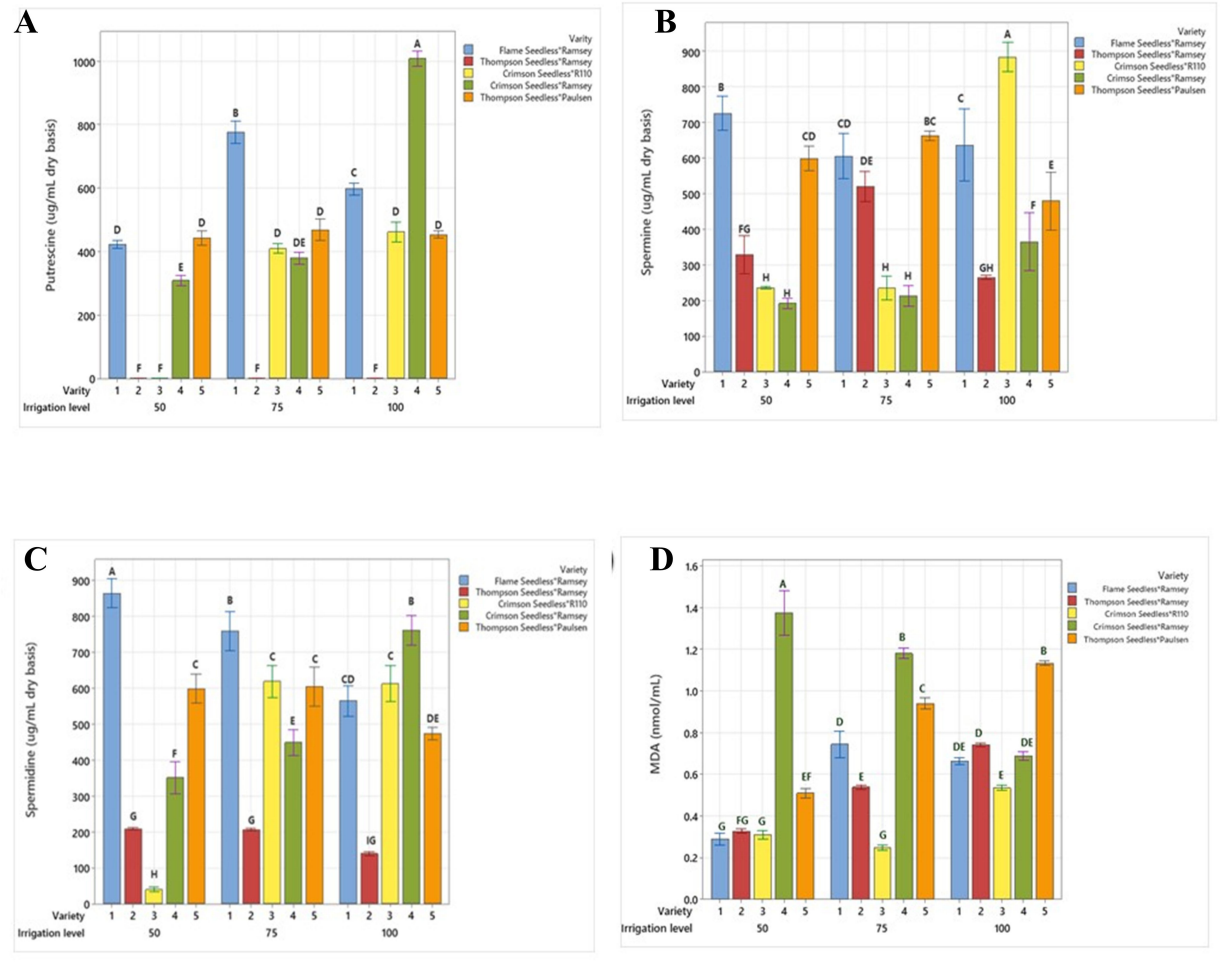
Comparison of **(A)** putrescine, **(B)** spermine, **(C)** spermidine and **(D)** MDA between five combinations of grafted grapevines (V1, V2, V3, V4, and V5) under three regimes of irrigation (100 %, 75 %, 50 % FC; 53 L, 40 L, 26 L, respectively), calculated on dry weight irrigation. (P ≤ 0.05).

A significantly higher level of putrescine (Put) was observed in V4 at 100% FC (1,000 µg mL^−1^) compared to other varieties (**Figure 4A**). Under the same irrigation level, the concentration in V1 was determined as 600 µg mL^−1^ and as 440 µg mL^−1^ in V3 and V5. In V1, the *Put* level increased sharply at 75% FC by 30%, while a subtle increase of 2.2% was observed in V5. In contrast, V4 and V3 exhibited reductions of 62% and 10%, respectively. At 50% FC, V5 did not exhibit any significant changes in concentration compared to 100% FC, while significant reductions of 72% in V1 and 25% in V4 were observed. V3 exhibited a significant decrease, and almost no *Put* was detected (**Table 4**).

**Table 4:** Summary of mean values (Irrigation: 100% FC, 75% FC, 50% FC and varieties: V1, V2, V3, V4, V5) and two-way ANOVA *p*-values (Irrigation, Graft and Interaction).

Response	100% FC	75% FC	50% FC	V1	V2	V3	V4	V5	P (Irrigation)	P (Graft)	P (Interaction)
Putrescine	504.14	406.61	234.82	598.7	0.0	290.5	565.4	454.7	<0.001	<0.001	<0.001
Spermidine	509.76	527.14	412.15	728.8	184.9	423.3	519.6	558.5	<0.001	<0.001	<0.001
Spermine	525.94	447.13	416.39	656.12	371.36	451.38	256.7	580.21	<0.001	<0.001	<0.001
Fructose	34.753	33.892	34.722	36.889	30.81	33.087	31.125	40.367	0.176 (NS)	<0.001	<0.001
Glucose	39.175	39.205	40.294	41.429	34.962	40.449	36.072	44.879	0.175 (NS)	<0.001	<0.001
MDA	0.7519	0.7295	0.5622	0.5656	0.5360	0.3635	1.0804	0.8604	<0.001	<0.001	<0.001
Catalase	7.280	11.680	17.000	11.933	9.067	8.733	11.733	18.467	0.189 (NS)	<0.001	0.012
SOD	13.360	16.640	53.480	28.133	21.800	25.133	26.000	38.067	0.120 (NS)	0.003	<0.001
RWC	94.2480	90.9280	84.8320	91.0067	89.780	90.8467	92.5533	85.8267	<0.001	<0.001	<0.001
POD	9.480	17.280	36.120	17.400	26.733	19.533	18.667	22.467	<0.001	0.666 (NS)	0.001
GR	7.920	9.720	19.120	10.867	9.733	12.533	14.400	13.733	<0.001	<0.001	0.016
APX	3.1160	4.7320	8.2880	4.3933	5.7467	4.5067	5.0333	7.2133	<0.001	<0.001	<0.001
Glycine Betaine	118.640	146.560	328.000	181.533	176.533	204.667	188.800	237.133	<0.001	<0.001	<0.001

The spermine (Spm) level in V2 was 260 µg mL^−1^ (100% FC), which significantly increased by 50% and 38.4% in 75% and 50% FC, respectively (**Figure 4B**). A similar trend was observed in V5, with a 500µg mL^−1^ concentration at 100% FC that increased by 36% and 20% at 75% FC and 50% FC, respectively. The Spm level was markedly reduced in V3 from 79% to 73%, while in V4, at 75 FC and 50 FC decrease of 43% and 46% was noticed, respectively, in comparison to 100% FC. Here, the Spm concentration was observed as 900 µg mL^−1^ in V3 and 500 µg mL^−1^ in V5. In V1 the Spm content was 640 µg mL^−1^ at 100% FC, dropping by 6.2% at 75% FC and significantly increasing by 22% at 50% FC.

The spermidine (Spd) level at 100% FC in V2 and V5 was 160 µg mL^−1^ and 480 µg mL^−1^, a 25% increase in concentration was observed at 75% FC, and no change occurred at 50% FC in both V2 and V5 (**Figure 4C**). In V3, the Spd concentration continuously dropped from 640 µg mL^−1^ (100% FC) to 600 µg/mL (75% FC), In V1, the Spd level showed significant continuous increases from 30% (75% FC) to 40% (50% FC) compared to 100% FC (600 µg mL^−1^). A reverse trend was observed in V4, where the Spd concentration decreased by 46.1% and 54% under irrigation at 75% and 50% FC, while at 100% FC, the concentration was determined as 780 µg mL^−1^ (**Figure 4C**).

### 3.2 Malondialdehyde

A significant difference was observed in the levels of MDA accumulation in V4 between the irrigation treatments. Increases of 71.4% and 97% were determined at 75% FC (1.2 nmoL mL^−1^) and 50% FC (1.38 nmoL mL^−1^), respectively, compared to 100% FC (0.7 nmoL mL^−1^) (**Figure 4D**). Grafts V2 and V5 exhibited a similar pattern of MDA accumulation at 75% and 50% FC, with reductions of 34.2% and 50% in V2 and 23.7% and 57.6% in V5 compared to 100% FC (0.76 nmoL mL^−1^ in V2 and 1.18 nmoL mL^−1^ in V5). At 100% FC, the MDA concentration in V1 was 0.68 nmoL mL^−1^ and subsequently rose by 11.76% with the water deficit at 75% FC. The concentration then significantly dropped by 55.8% at 50% FC. In V3, a 52% and 40% decline was observed at 75% and 50% FC, respectively, compared to 100% FC (**Table 4**).

### 3.3 Sugars

**Figure 5:**
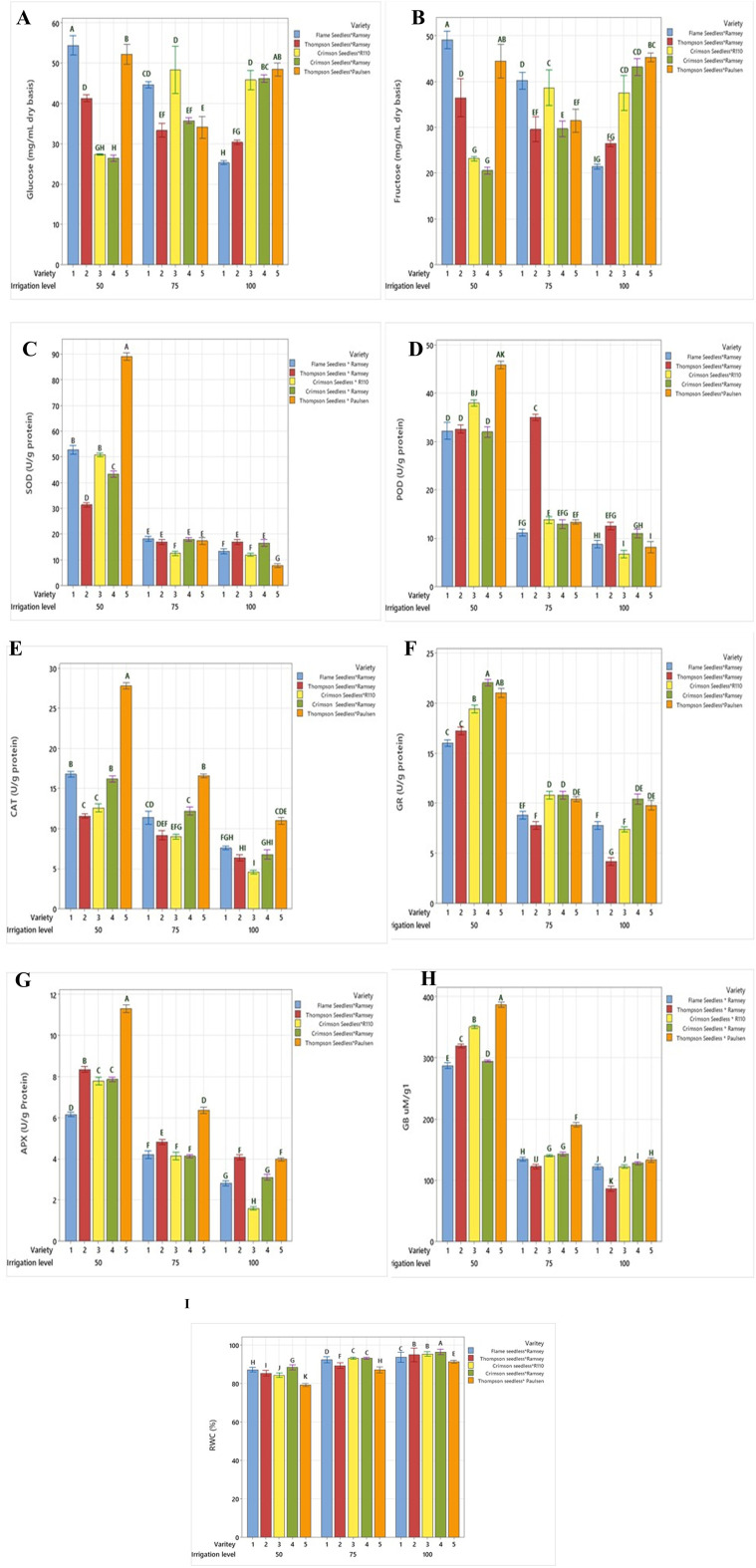
Comparison of **(A)** glucose, **(B)** fructose, **(C)** SOD, **(D)** POD, **(E)** CAT, **(F)** GR, **(G)** APX, **(H)** GB and **(I)** RWC between five combinations of grafted grapevines (V1, V2, V3, V4, and V5) under three regimes of irrigation (100 %, 75 %, 50 % FC; 53 L, 40 L, 26 L, respectively), calculated on dry weight irrigation. (P ≤ 0.05).

The accumulation of leaf-soluble sugars varied among the graft combinations. In V1, V2, and V4, the glucose sugar level was 21, 26 and 43 mg g^−1^ DW, respectively, at 100% FC (**Figure 5A**).

The glucose sugar content at 75% and 50% FC increased significantly by 85.7% and 133% in V1, respectively. In V2, the increase was 19% at 75% FC and 76.9% at 50% FC, while in V4, reductions of 30% and 53% were observed. Graft V3 exhibited a 19% rise at 75% FC and a 76.9% decrease at 50% FC compared to 100% FC. In V5, a significant decrease of 30.4% was observed at 75% FC, while there was no significant difference seen between 100% FC and 50% FC. The accumulation of leaf fructose significantly increased in V1 and V2 between 75% FC and 50% (from 80% to 116% and 9.7% to 22.5%, respectively), compared to 100% FC (**Figure 5B**). Grafts V3 and V4 showed a reduced accumulation of fructose for 75% and 50% FC. These two grafts exhibited a similar fructose level at 100% FC (46 mg g^−1^), which decreased at 75% and 50% FC. The decline in fructose content in V3 was not significant at 75% FC (2.2%), yet it dropped significantly to 41.3% at 50% FC. Reductions of 23.9% to 43.4% were determined in V4. In V5, the fructose concentration at 100% FC was 49 mg g^−1^, while at 75% FC, a decrease of 46.9% occurred, and an 8.1% significant increase was observed at 50% FC compared to 100% FC (**Table 4**).

### 3.4 Antioxidant enzymes

SOD, POD, CAT, GR and APX activities progressively increased with drought stress under 75% FC and 50% FC compared to 100% FC for the five grafted varieties (**Table 4**).

SOD activity under 75% FC and 50% FC significantly increased by 25% to 225% in V1, 8.3% to 316% in V3, 11.1% to 133% in V4, and 150% to 1025% in V5, respectively, compared to 100% FC (**Figure 5C**). The enhancement in SOD activity in V5 was significantly higher compared to that of the other grafts at all drought stress levels. In V2, the SOD activity under 100% FC and 75% FC was 18 µg^−1^, which further increased by 66.6% under 50% FC.

POD activity under 75% FC and 50% FC significantly increased by 9% to 181.8% in V1, 36.4% to 245.5% in V3, 33.3% to 158.3% in V4, and 77.7% to 400% in V5, respectively, compared to 100% FC (**Figure 5D**). In V2, POD activity under 75% FC increased by 133.3% and decreased by 113.3% under 50% FC.

CAT activity under 75% and 50% FC significantly increased by 29.4% to 88.8% in V1, 80% to 160% in V3, 71.4% to 128.5% in V4, and 41.6% to 125% in V5, respectively, compared to 100% FC (**Figure 5E**). In V2, CAT activity under 75% FC increased by 85.7% and decreased by 71.4% under 50% FC.

GR activity under 75% FC and 50% FC significantly increased from 12.5% to 100% in V1, 60% to 240% in V2, 33% to 111% in V3, 9% to 100% in V4, and 8.3% to 833% in V5, respectively (**Figure 5F**). The rise in GR activity at 50% FC was higher in all the grafts compared to the control treatment (100% FC) and deficit irrigation treatment (75% FC). APX activity in the leaf increased as the irrigation treatment progressed from 75% to 50% FC compared to 100% FC. A significant rise was observed in all the varieties (**Figure 5G**), with increases of 55.7% to 134.8% in V1, 10% to 105% in V2, 127.8% to 333.3% in V3, 46.4% to 214.3% in V4, and 55% to 177.5% in V5 between 75% FC and 50% FC, respectively.

### 3.5 Glycine betaine

A significant increase in the percentage of glycine betaine (GB) was observed at 50% FC compared to 100% FC, with V1 exhibiting the highest percentage (**Figure 5H**). GB content in the graft leaves showed an increasing trend with the water-deficit treatments (75% and 50% FC). An extremely significant increase was observed at 50% FC compared to 100% FC, with values increasing from 8.3% to 141.77% in V1, 33.3% to 222.2% in V2, 16.7% to 191.7% in V3, 12% to 140% FC in V4, and 46.2% to 200% in V5 at 75% FC and 50% FC, respectively (**Table 4**).

### 3.6 RWC

Graft V1 demonstrated a drop in RWC by 1% and 6.5% at 75% FC and 50% FC, respectively, while V2, V3, and V4 exhibited reductions of 4.3% and 85% at 75% FC and 50% FC, respectively (**Table 4**). The RWC decreased markedly in V5 from 75% FC (5.4%) to 50% FC (12%) (**Figure 5I**). These findings imply that the grapevine grafts reacted differently to water-deficit treatments. The results emphasize the importance of selecting the right graft to assist grapevine resistance to water stress and ensure maximum growth and yield under a variety of circumstances.

## 4 Discussion

### 4.1 Malondialdehyde

The levels of MDA are elevated following an increase in free radical production in plants. MDA is a product of the peroxidation of membrane lipids in cells under drought in response to ROS accumulation (Hasanuzzaman et al., 2020). The MDA content in plants is directly proportional to the extremity of drought in plants and is therefore referred to as a marker of oxidative lipid damage to reflect the response of plants to stress. Morales et al. (2021) reported that the greater the plant damage, the higher its MDA content. In this study, we observed a rise in MDA accumulation in graft V4 by 71.4% and by 97% in 75% FC (1.2 nmoL mL^−1^) and 50% FC (1.38 nmoL mL^−1^), respectively, compared to 100% FC (0.7 nmoL mL^−1^). This indicates the sensitivity of the plasma membrane permeability to disruption, leading to the extravasation of the cell contents. The decrease of 34.2% and 50% in V2 and 23.7% and 57.6% in V5 compared to 100% FC (0.76 nmoL mL^−1^ in V2 and 1.18 nmoL mL^−1^ in V5) clearly show the combative role played by lipid peroxidation. Reduced MDA content in cells is linked to a plant’s increased capacity to fight drought stress. This allows it to assess the degree of oxidative stress-induced damage by observing changes in MDA production in plant cells.

Total antioxidant capacity (T-AOC) is considered a strong indicator of the overall antioxidant level of plants based on various antioxidant enzymes and antioxidants (Saddique et al., 2020). Together with the concentration of MDA, they are significant indicators of the drought resistance of plants. The accumulation of MDA due to lipid peroxidation, the main effect of oxidative damage, has also been observed during the imposition of other abiotic stresses in sunflower (Bailly et al., 1996). The MDA level provides evidence of a higher lipid peroxidation coinciding with LOX activity in stressed plants, as reported by Lima et al. (2002) in *Coffea canephora* and by Sofo et al. (2004) in olive trees. The observed high degree of lipid peroxidation can produce lipid derivatives acting as secondary messengers capable of activating drought-stress-associated genes by specific transcription factors, which can start the desiccation response of plants (Ingram and Bartels, 1996). Increased ROS generation and MDA content have negative effects on metabolic activities that play key roles in boosting the immune system of plants to resist different stress conditions (Hasanuzzaman et al., 2020).

The grafting of wild grapevines Dakh and Fatati onto commercial varieties of grapes can metabolize reduced levels of MDA content. This may be attributed to a higher RWC maintained in the tissues of the grafted varieties, even at higher levels of drought stress (Nazir et al., 2022). This conforms to the observed MDA content in the grafted grapevines in the present study under extreme levels of heat and drought. A significant increase in the content of soluble sugars was observed across the grapevine grafts tolerant to drought stress, which is concurrent with the increase in MDA content and the potential role of soluble sugars as osmotically active solutes to mediate osmoregulation and reduce water-deficit damage. Bacelar et al. (2006) found the levels of sugars to increase in three olive cultivars subjected to water deficit. Previous research also observed the concentration of soluble sugars in strawberry leaves subjected to drought stress to increase with the severity and duration of drought stress (Sun et al., 2015).

### 4.2 Polyamines

Endogenous levels of polyamines have been ascribed as regulating growth under moisture stress, essentially acting as intracellular messengers to mediate physiological and biochemical responses. Spm, Spd, and Put, which are the diamine obligate precursors, have great relevance in growth regulation processes under abiotic stress conditions. Davies (2010) reported on the specific roles of polyamines in rice subjected to drought. In higher plants, Put can be directly synthesized from ornithine via ornithine decarboxylase and/or indirectly through arginine via arginine decarboxylase. The significant increase in Put at 75% FC in V1 by 30% and the marginal increase of 2.2% in V5 are evidence of the stress response in grape grafts, in conformity with the above findings. The biosynthesis of Put mediated through arginine was observed in grapevine in our study, with a sharp increase at 75% FC irrigation in V5. In contrast, the 62% decrease in Put in V4 is related to the decrease in arginine levels under 75% and 50% FC. Furthermore, the Put levels under stress in V1 indicate that the increase in *Put* biosynthesis is primarily the result of increased arginine biosynthesis via arginine decarboxylase in the regulatory mechanism, as observed by Yang et al. (2007) in rice plants.

Drought-tolerant grape grafts had higher free Spm and free Spd levels in the leaves (**Figures 4B, C**) compared to drought-susceptible grafts under water stress. The V1, V2, and V5 grafts exhibited an increase in the content of Spm and free Spd, while V3 and V4 exhibited sensitivity to exposure to moisture stress, with low levels of Spm and Spd. Higher Spm and Spd generally adhere to the negative charges in cellular components and thereby equilibrate the membrane integrity under stress conditions, as reported by Kasukabe et al. (2004) and Ma et al. (2005). Furthermore, PAs have been associated with antioxidant enzyme activities such as superoxide dismutase, catalase, and peroxidase, functioning as free radical scavengers. Overexpression of Spd synthase enhances tolerance to a variety of environmental stresses and upregulates the expression of various stress-regulated genes, as shown in transgenic *Arabidopsis thaliana* (Kasukabe et al., 2004). Capell et al. (2004) reported the modulation of the polyamine biosynthetic pathway in transgenic rice, conferring tolerance to drought stress. Therefore, ROS scavenging, membrane integrity stabilization and gene overexpression properties may be involved in the specific stress-reducing roles of Spm and Spd in grape grafts, including the observation in higher plants.

### 4.3 Sugars

Plants have adapted to abiotic stressors by altering their metabolism, which results in the build-up of various organic solutes, including sugars. According to Sun et al. (2015), hydroxyl molecules such as sucrose, polyhydric alcohols, and oligosaccharides, are among the compatible solutes that have an impact on osmotic adjustments via., the accumulation of sugars (apart from other macro-molecules). Cellular sugar status is maintained optimally during normal growth conditions but is impacted negatively during various environmental perturbations such as drought stress, as reported in grapevine grafts (Kaur et al., 2021). Drought is a critical unfavourable environment that limits the photosynthetic fixation of carbon into sugars (Poonam et al., 2016). Sugars affect transport by lowering the cellular osmotic potential. Kaur et al. (2021) reported that the transport of cellular sugar is facilitated by a specific set of proteins known as sugar transporters. These transporter proteins are key determinants of the influx/efflux of various sugars and their metabolite intermediates that support plant growth and developmental processes (Li et al., 2021). The observed increase in sugar content in V1 and V5 points to sugars accumulated in source tissues that are transported to sink tissues via the sugar transporters. The transportation of sugars ensures their distribution throughout the plant system, which facilitates their utilization as osmo-protectants and energy sources throughout the plant to combat stress, as observed in grapevines.

The elevated levels of glucose monosaccharide content in 75% and 50% FC (85.7% to 133% in V1, 19% to 76.9% in V2; 30% and 53% reduction in V4, respectively) are an indication of the graft response to drought challenges and its tolerance capabilities. The high levels of monosaccharide sugars such as trehalose, sucrose, and raffinose prevent protein denaturation, stabilize membranes, and act as ROS scavengers. Our results are consistent with the findings of Almeida et al. (2007).

Starch depletion in grapevine leaves was reported by Patakas and Noitsakis (2001), whereby the concentrations of soluble sugars increased while starch concentrations decreased (Hunter et al., 1994). The glucose, mono-, di- and oligosaccharides, which were observed to accumulate in the grafts under deficit irrigation, could act as osmo-protectants. Apart from this role, soluble sugars also act as signalling molecules to modulate the sensitivity of plants to evoke cell responses. The exogenous application of various sugar molecules (e.g., trehalose, mannitol, and sorbitol) may also elicit tolerance mechanisms against unrelated abiotic stresses, including drought stress (Ahmad et al., 2019). In response to drought stress, the glucose, fructose, and sucrose content of leaf samples in V5 increased significantly, while in V4, V3, and V2, there was a significant decrease in sugar molecules. Furthermore, glucose and fructose significantly decreased under drought stress in V1. The active synthesis metabolism of sugars and proline (as noticed in the study, Krishankumar et al., unpublished) in tolerant grafts was dramatically enhanced in shoots, which may have improved the ROS detoxification ability, osmotic adjustment, membrane integrity, and ultimately drought tolerance. This is consistent with the findings of Marcińska et al. (2013).

### 4.4 Catalase activity

The significant increase in CAT activity under 75% and 50% FC irrigation in V1, V3, V4, and V5 points to the combative mechanism of grape grafts under environmental perturbations. The imposed stress coincided with an increase in the production of ROS and reactive nitrogen species, resulting from an imbalance between their production and scavenging. The tolerance of the drought-tolerant rootstock–scion relationship, in which the scions are sensitive to drought, is a noteworthy characteristic of all the grafts. Insensitive plants that undergo CAT activity inhibition experience increased photorespiration due to stomatal closure, a decrease in intercellular CO_2_, increased RUBISCO oxygenation, and consequently, boosted H_2_O_2_ release by glycolate oxidase in the peroxisome. As CAT is necessary to scavenge the produced H_2_O_2_, inhibiting it during a drought will disrupt the balance of redox and ROS. This is consistent with the study of Gechev et al. (2013), whereby CAT activity was inhibited to express sensitivity to drought, most likely by the enhanced release of H_2_O_2_, which impacted redox reactions and ROS homeostasis. Therefore, an increase in CAT indicates drought tolerance. The observed CAT activity and drought tolerance induced in the sensitive scions (table grape varieties) by the drought-tolerant rootstocks in the current study highlight the importance of finding tolerant rootstocks that enhance oxidative activities under difficult environmental conditions in arid regions, facilitating sustainable grapevine cultivation. The results are consistent with those of Nazir et al. (2022) on wild grapevine species and their hybrid genotypes, which were utilized as rootstock to make vine production sustainable under abiotic and biotic stresses due to the presence of inherited genetically tolerant traits.

The genetic tolerance of the rootstock and the transfer of certain proteins in the current study, in inducing tolerance to grafts in growth (**Figures 2A-D** at the field establishment stage) and production (**Figure 2E**) have been established in unequivocal terms. Drought conditions require a high capacity of detoxifying enzymes, including APX and CAT, to reduce ROS formation. The activation of these two primary scavengers is stronger in tolerant species than in their sensitive counterparts (Laxa et al., 2019), as exhibited by the different grape grafts tested for CAT activity in this study (**Figure 5E**). The expressions of specific CAT genes that are upregulated following drought/desiccation, as reported by Laxa et al. (2019), may have influenced the enhanced activity of CAT in all the grafts.

### 4.5 Superoxide dismutase activity

A significant increase in SOD activity in different grafts (by 25% to 225% in V1, 8.3% to 316% in V3, 11.1% to 133% in V4, and 150% to 1,025% in V5) was revealed in the study. This is evidence of the critical role of SOD induced by the rootstocks. In V2, the SOD activity under 100% and 75% FC was further increased by 66.6% (18 µg g^−1^) under 50% FC. This points to the critical interplay between the rootstock and scions upon exposure to induced drought. This could be attributed to a reprogramming of metabolism (Zhang et al., 2014) and the activation of the antioxidant system (Ajithkumar and Panneerselvam, 2014; He et al., 2017), as evidenced in this study by the response of SOD activity with remarkably upregulated activity of the enzyme following upon inducing drought. Higher levels of SOD and CAT activities were observed when commercial scion varieties Kings Ruby and Flame Seedless were grafted on relatively tolerant wild grapevines Dakh and Fatati (Nazir et al., 2022). This may be because wild grapevines upregulate genes, which increases the activities of SOD and CAT enzymes in grafted scion varieties. In addition, Haider et al. (2018) reported the expression of the “CAT1,” “SOD,” and “Cu-Zn” genes encoded by the ROS-scavenging system. A class of metallo-enzymes known as SODs catalyzes the dismutation of two molecules of O2 ^●−^ into H_2_O_2_ and molecular oxygen. The activation of SOD isoforms (Mn-SOD, Fe-SOD, and Cu, Zn-SOD) is interpreted as a measure to counteract O_2_ accumulation in diverse cell compartments under drought, as shown in *Arabidopsis* (Jung, 2004), blue grass (Fu and Huang, 2001), citrus (Zandalinas et al., 2017), and *Coffea canephora* (Lima et al., 2002). SOD is an essential part of the ROS-scavenging mechanism by minimizing the reaction of O_2_^●−^ with, for example, NO (Nitric oxide) to form ONOO^−^, unsaturated fatty acids for peroxidation or with proteins (Wang et al., 2018). In line with this assumption, transgenic plants overexpressing Cu. Zn-SOD are more tolerant to drought stress (Wu et al., 2016).

Membrane damage is a common occurrence under prolonged drought, where the drought tolerance mechanism deals with membrane damage due to the excessive cellular generation of ROS. Wild grapevines trigger the enhanced activities of SOD to dismutase free oxygen radicals into H_2_O_2_. Following this, the CAT enzyme converts H_2_O_2_ into water molecules and maintains an oxidative equilibrium. The same concept applies in the present study with drought-tolerant rootstocks to induce tolerance under hostile environmental conditions induced by varied irrigation levels.

### 4.6 Peroxidase

POD activity increased significantly in V1, V3, V4 and V5 grafted grapevines at the 50% FC water-deficit treatment (D’Iaz-Vivancos et al., 2016), in V2, which declined under 50% FC but was higher than the observed content at 100% FC and lower than that at 75% FC. Grafting has been widely utilized to increase scion cultivars’ resistance to oxidative stress and to maintain the productivity of grafted plants in drought-stressed environments (Yang et al., 2022). The CAT enzyme activity can inhibit the excessive generation of ROS. However, due to the strong interplay of CAT with APX and POD, the drought-protective rootstocks of grapevines upregulate the APX and POD enzymes to reduce the toxic effect of H_2_O_2_, which must be further converted to O_2_ and H_2_O to nullify the effects (Abid et al., 2018). This phenomenon has been reported by D’Iaz-Vivancos et al. (2016) with the wild grape rootstocks Dakh, Fatati, and Toran. The improved upregulation of transcriptional responses related to enzymatic activities such as APX may be related to the strong transcription episodes that could be effectively upregulated by tolerant wild grapevines through the perception of signals and electron transportation generated from an imbalanced redox state, as reported by Santos et al. (2019). The same effect may have played a role in the grafting of rootstocks such as Ramsey and Paulsen with scions susceptible to soil moisture deficits to induce tolerance. The genetic accentuation of different wild grapevines to upregulate the expression of genes related to antioxidant enzymes was also corroborated by Balfagón et al. (2018). Thus, the enzymatic responses of POD in wild grapevines may be one of the key indices against different levels of tolerance exhibited by rootstock Ramsey or Paulsen, for example, the use of Ramsey in the V2 graft.

### 4.7 Glutathione reductase

GR acts as a significant player in desiccation tolerance in grapevine grafts exposed to reduced water field capacities (Vašková et al., 2023). Research by Toldi et al. (2009) is consistent with this, indicating a strong antioxidant status as a prerequisite for desiccation tolerance in the resurrection of plants exposed to drought. Based on this, glutathione is suggested to be an important player in the dehydration responses of plants. GR activity is enhanced in many plant species under various abiotic stresses, such as exposure to drought, heavy metals, salinity, ultraviolet radiation, and cold temperatures. In addition, several stress-tolerant plants display high GR activity (Amsellem et al., 1993). GR is a homodimer enzyme that is essential for the function of the glutathione/ascorbate cycle. It also provides the GSH required for MG detoxification (Marty et al., 2009). Each isoform contributes to total GR activity (Kunert and Foyer, 2022). Therefore, the functional polymorphism expressed by GR is evident in grape grafts when different rootstocks are combined with scions and subjected to different field capacities of irrigation. The small enzymatic contribution of peroxisomal GR may be an important signalling indicator during drought (Romero-Puertas et al., 2006). Under catalase-deficient conditions (cat2 mutant impaired in H_2_O_2_ removal), GR1 is needed to respond to stress situations that generate high levels of H_2_O_2_ (Mhamdi et al., 2010). Transgenic approaches to manipulate GR activity in plants also confirm that elevated GR activity plays a prominent role in improving tolerance to the oxidative stress caused by a variety of abiotic factors due to efficient ROS-scavenging capacity (Gill et al., 2013).

### 4.8 Ascorbate peroxidase

The significant upregulation in APX under different irrigation regimes indicates its strong antioxidant capacity to mitigate the toxic effect of free radicals. The results point to the strong enzyme activity in all the grafts when exposed to deficit irrigation. This is in line with the findings of D’Iaz-Vivancos et al. (2016), who reported that the CAT enzyme can dismutase excessive ROS generation under drought stress. However, as CAT, APX, and POD activities are strongly correlated, wild grapevines upregulate APX and POD enzymes to reduce the harmful effects of H_2_O_2_ (Hasanuzzaman et al., 2020). The grafting of commercial scion cultivars Kings Ruby and Flame Seedless on highly tolerant wild grapevines Dakh, Fatati, and Toran resulted in an enhanced rate of enzymatic activity, particularly for APX (Nazir et al., 2022). Similarly, in our study, Thompson seedless grafted on Paulsen and Ramsey expressed excessive enzymatic activity while other grafts showed moderate yet significant enzyme activity at different deficit irrigation levels. This increased level of enzymatic activities may be associated with the strong transcription responses that could be upregulated effectively by Paulsen and Ramsey. This was also reported in relatively tolerant wild grapevines via signals and electron transportation that may result from the imbalanced redox state (Santos et al., 2019) in citrus Rangpur Santa plants compared to drought-susceptible citrus Sunki Maravilha. The upregulation of enzyme activity as a transcriptional response and the genetic accentuation of different wild grapevines to upregulate the expressions of different genes were also corroborated by Balfagón et al. (2018). In citrus drought-tolerant Carrizo rootstock, the expression of an additional APX1 gene that upregulates the activities of cytosolic APX and subsequently helps the grafted plants to acclimatize under drought stress conditions (Zandalinas et al., 2017) could be applied in grape grafts to upregulate the enzyme. Grafts V1 Flame seedless × Ramsey, V3 Crimson seedless × R110, and V4 Crimson seedless × Ramsey also exhibited the upregulation of APX, which strongly indicates the rootstock–scion interaction in the regulation of the APX. The optimal growth of grafted scion varieties generally associated with minimal drought damage may be linked to the increased rate of enzymatic activity through the relatively drought-tolerant grape rootstocks. This can efficiently upregulate the cellular activities and subsequently reduce the production of ROS in the shoots and leaves of the scions (Santos et al., 2020).

### 4.9 Glycine betaine

Similar to sugars or proline, organic osmolyte GB has a significant positive relation with enzymatic activities under drought stress, as observed in grafts involving drought-tolerant rootstocks such as Ramsey × TS, Paulsen × TS, and R110 × Crimson seedless at 50% FC irrigation (**Figure 5F**). This is linked to the behaviour of relatively drought-tolerant wild grapevines Dakh and Fatati to upregulate drought-related enzyme activities (Nazir et al., 2022), which may have enhanced the protection of osmolyte synthetization from suppression and the chlorophyll degradation in grafted commercial scion varieties Kings Ruby and Flame Seedless (Shaw et al., 2002). The findings indicate the enhanced activities of drought-responsive enzymes (**Figures 5C–G**) with the field morphology of grafted vines during the establishment stage. This confirms the ability of stable photosynthetic pigments in the face of drought challenges to combat stress (**Figures 2A–D**). Under 75% FC irrigation, the grafts V5, V4, and V3 continued to significantly upregulate GB to combat stress, which may be attributed to the ability of the rootstock to induce tolerance to scions. This agrees with findings on drought-tolerant citrus Mexican lime rootstock, which played a key role in improving the production of osmolytes in the grafted citrus Kinnow Mandarin scion variety by maintaining the chlorophyll content under drought-stress conditions (Shekafandeh et al., 2019) and in grafted grapes (Krishankumar et al., 2025). Moreover, wild-originated grapevine R110 rootstock significantly increased the accumulation of organic solutes in grapevines grafted on superior seedless cultivars, as observed in this study (**Figure 5G**).

### 4.10 Relative water content

Leaf relative water content (RWC) is an important indicator of plant water status. It has been shown that the RWC decreases in response to drought stress (Fu and Huang, 2001; Shaw et al., 2002). The relative water content (RWC) of grafted plants under drought stress can exhibit varied responses depending upon specific graft combinations and the physiological mechanisms involved. In the present study, the RWC was maintained steadily under different field capacities, thus exhibiting tolerance to drought (Fig.10). The present study indicated that grafting could enhance drought tolerance by maintaining the balance between water supply to the leaf tissue and transpiration rate (Vašková et al., 2023). In our study, we discovered that grafted grapevine varieties V1, V2, V3, V4 and V5 showed reduction in RWC under 75% FC and 50% FC, suggesting that certain graft combinations can effectively reduce water loss and enhance drought resilience (Jiao et al., 2023).

Rahmani et al. (2023) noted that while drought-tolerant cultivars could maintain higher RWC levels, the degree of maintenance varied significantly among different cultivars, indicating that not all grafted combinations are equally effective in preserving plant water status under drought stress. Conversely, in grafted grapes it was observed that the RWC showed a significant decline among the grafted varieties with progressive increase in induced stress, however the percentage of decrease in RWC varied among the varieties as seen in our study. Moreover, the physiological responses of grafted plants to drought stress can also be influenced by the rootstock’s ability to regulate water uptake and osmotic balance.

Chen et al. (2020) demonstrated that different rootstocks could modulate the drought tolerance of grafted poplars by affecting root-to-shoot signaling, which in turn influenced RWC and overall plant health under drought conditions. This aligns with the findings that grafting could enhance antioxidant enzyme systems in leaves, which are crucial for maintaining cellular water status during drought stress (Liu et al., 2012). Under drought stress situations due to the production of antioxidant enzymes there is a decrease in oxidative because of scavenging of ROS. This leads to the maintenance of RWC which is balancing leaf water potential and the transpiration loss of water. For example, CAT converts antioxidant enzyme which converts H_2_O_2_ to H_2_O in peroxisomes and lysosomes (Kaur et al., 2009) indicating the scavenging of free radicals.

In summary, the relative water content of grafted plants under drought stress can vary greatly depending on the choice of rootstock, the specific plant species and the physiological mechanisms involved. While some graft combinations show improved water retention and drought tolerance, others may not show significant benefits, highlighting the importance of understanding the interactions between grafted plants and their environment.

## 5 Conclusion

This study shows that grafted grapevines respond biochemically to drought by upregulating antioxidant enzymes and compounds, protecting cellular integrity. Physiologically, water stress did not affect relative water content or membrane stability in the five grapevine varieties tested. Biochemically, drought increased MDA levels, particularly in Crimson seedless × Ramsey, while antioxidant enzymes like SOD, POD, APX, GR, and CAT rose, reducing ROS and supporting cell health. In drought-tolerant rootstocks (Ramsey, Paulsen, and R110), sugar levels linked positively with enzyme activity, acting as osmo-protectants and signaling molecules that stabilized proteins and membranes. Sugar metabolism in these grafts enhanced ROS detoxification, osmotic balance, and drought resilience. Polyamines varied, with some grafts maintaining high Spm and Spd under stress, supporting membrane integrity. This modulation of sugars and PAs in grafted grapevines appears critical for drought tolerance. The findings underscore the capacity of grafted grapevines to adapt to drought stress through the upregulation of antioxidant enzymes, MDA, PAs, and sugars. Enhanced sugar metabolism in drought-tolerant grafts emerges as a critical factor in their resilience to water stress. This study contributes to the understanding of the biochemical mechanisms underpinning drought tolerance in grapevines, highlighting the importance of sugar modulation in plant responses to environmental stressors.

## Data Availability

The original contributions presented in the study are included in the article/**Supplementary Material**. Further inquiries can be directed to the corresponding authors.
