# Supplementary material for: Influence of different scion-rootstock combinations on sugars, polyamines, antioxidants and malondialdehyde in grafted grapevines under arid conditions
Source: Front Plant Sci. 2025 Jun 27;16:1559095. doi: 10.3389/fpls.2025.1559095 (PMC12246978; doi:10.3389/fpls.2025.1559095)
Supplement: Supplementary file 1 [file Table1.docx]

**Supplementary Table(s)**

**Supplementary Table 1:** Content of GB, CAT, SOD, RWC, POD, GR, APX, MDA, putrescine, spermidine, spermine, glucose, fructose in the leaves of graft varieties (V1, V2, V3, V4, V5) subjected to three levels of irrigation treatment (100% FC, 75 FC and 50 FC). Values are presented as means ± SE (n = 5) corresponding to the figures.

| **IRRIGATION LEVELS**  *****  **VARIETIES** | **100% FC** | | | | | **75% FC** | | | | | **50% FC** | | | | | |
| --- | --- | --- | --- | --- | --- | --- | --- | --- | --- | --- | --- | --- | --- | --- | --- | --- |
|  | **V1** | **V2** | **V3** | **V4** | **V5** | **V1** | **V2** | **V3** | **V4** | **V5** | **V1** | **V2** | **V3** | **V4** | **V5** |  |
| **Glycine Betaine (GB)** | 122.2±0.86J | 86.8±0.8K | 122.6±0.51J | 128.2±0.49I | 133.4±0.68H | 134.8±0.58H | 123±0.71J | 140.6±0.4G | 143.4±0.68G | 191±0.71F | 287.6±0.93E | 319.8±0.58C | 350.8±0.58B | 294.8±0.37D | 387±0.84A |  |
| **Catalase (CAT)** | 7.6±0.81FGH | 6.4±0.58HI | 4.6±0.32I | 6.8±0.49GHI | 11±0.24CDE | 11.4±0.24CD | 9.2±0.4DEF | 9±0.24EFG | 12.2±0.58C | 16.6±0.45B | 16.8±0.37B | 11.6±0.24C | 12.6±0.51C | 16.2±0.37B | 27.8±0.37A |  |
| **Superoxide Dismutase (SOD)** | 13.4±0.51F | 17±0.45E | 12±0.32F | 16.6±0.68E | 7.8±0.37G | 18.2±0.49E | 17±0.45E | 12.6±0.4F | 18±0.32E | 17.4±0.68E | 52.8±0.86B | 31.4±0.4D | 50.8±0.37B | 43.4±0.6C | 89±0.71A |  |
| **Relative water content (RWC)** | 93.64±0.13C | 94.82±0.26B | 95.22±0.11B | 96.3±0.15A | 91.26±0.1E | 92.24±0.08D | 89.22±0.1F | 93.1±0.04C | 93.04±0.05C | 87.04±0.18H | 87.14±0.06H | 85.3±0.11I | 84.22±0.1J | 88.32±0.15G | 79.18±0.09K |  |
| **Peroxidase (POD)** | 8.8±0.37HI | 12.6±0.4EFG | 6.8±0.37I | 11±0.45GH | 8.2±0.58I | 11.2±0.37FG | 35±0.32C | 13.8±0.37E | 13±0.45EFG | 13.4±0.24EF | 32.2±0.86D | 32.6±0.4D | 38±0.32BJ | 32±0.55D | 45.8±0.37AK |  |
| **Glutathione Reductase (GR)** | 7.8±0.37F | 4.2±0.37G | 7.4±0.24F | 10.4±0.51DE | 9.8±0.49DE | 8.8±0.37EF | 7.8±0.37F | 10.8±0.37D | 10.8±0.37D | 10.4±0.24DE | 16±0.32C | 17.2±0.37C | 19.4±0.4B | 22±0.32A | 21±0.45AB |  |
| **Ascorbate Peroxidase (APX)** | 2.82±0.06G | 4.08±0.06F | 1.6±0.04H | 3.1±0.07G | 3.98±0.04F | 4.2±0.09F | 4.82±0.07E | 4.14±0.09F | 4.14±0.04F | 6.36±0.08D | 6.16±0.05D | 8.34±0.07B | 7.78±0.1C | 7.86±0.05C | 11.3±0.09A |  |
| **MDA** | 0.66375±0.0169DE | 0.742±0.00793D | 0.5339±0.0126E | 0.6869±0.0198DE | 1.13275±0.0114B | 0.743±0.0628D | 0.5378±0.0114E | 0.2478±0.0132G | 1.1797±0.025B | 0.939±0.0261C | 0.29±0.0275G | 0.3282±0.00966FG | 0.30875±0.0207G | 1.3745±0.107A | 0.5095±0.0238EF |  |
| **Putrescine** | 597.1±19.3C | 0±0F | 461.9±31.2D | 1008.1±23.1A | 453.6±10.3D | 776.3±34.3B | 0±0F | 409.5±14.9D | 379.3±19.2DE | 467.9±34.2D | 422.6±11.7D | 0±0F | 0±0F | 308.8±16.7E | 442.7±23.4D |  |
| **Spermidine** | 564.1±21.4CD | 139.63±2.91G | 611.8±24.8C | 759.9±21B | 473.32±8.24DE | 759±27.2B | 206.26±1.92G | 617.9±22.3C | 448.5±18E | 604±27.4C | 863.2±19.9A | 208.72±2.08G | 40.18±3.19H | 350.4±22.6F | 598.2±20C |  |
| **Spermine** | 636.762±33.9C | 264.59±1.97GH | 883.709±13.7A | 365.323±26.9F | 479.311±27.3E | 605.4±21.2CD | 520.4±14.1DE | 234.5±11.3H | 212.83±9.56H | 662.53±4.49BC | 726.187±16.1B | 329.12±17.9FG | 235.94±1.35H | 191.949±4.6H | 598.777±11.5CD |  |
| **Fructose** | 21.46±0.176H | 26.474±0.217FG | 37.472±1.26D | 43.122±0.628BC | 45.236±0.331AB | 40.178±0.615CD | 29.532±0.906EF | 38.62±1.28D | 29.686±0.565EF | 31.448±0.833E | 49.028±0.637A | 36.424±1.39D | 23.174±0.155GH | 20.568±0.247H | 44.416±1.23B |  |
| **Glucose** | 25.314±0.231G | 30.346±0.247FG | 45.756±1.19CD | 46.084±0.469CD | 48.376±0.814BC | 44.63±0.379CD | 33.306±0.843EF | 48.28±2.92BC | 35.724±0.356E | 34.08±1.36EF | 54.344±1.17A | 41.234±0.438D | 27.306±0.0792G | 26.408±0.4G | 52.18±1.23AB |  |

Different letters denote significant differences between means according to the Tukey HSD post hoc.
